# Native Microbiome Members of *C. elegans* Act Synergistically in Biosynthesis of Pyridoxal 5′-Phosphate

**DOI:** 10.3390/metabo12020172

**Published:** 2022-02-11

**Authors:** Orçun Haçariz, Charles Viau, Xue Gu, Jianguo Xia

**Affiliations:** 1 Institute of Parasitology, McGill University, Montreal, QC H9X 3V9, Canada; orcun.hacariz@mail.mcgill.ca (O.H.); charles.viau@mail.mcgill.ca (C.V.); xue.gu@mcgill.ca (X.G.); 2 Department of Animal Science, McGill University, Montreal, QC H9X 3V9, Canada

**Keywords:** *C. elegans*, microbiome, vitamin B6, pyridoxal 5′-phosphate, glutamine, LC-MS/MS, metabolomics

## Abstract

The roles of the healthy microbiome on the host and the relationships between members of the microbiome remain to be fully characterized. Due to the complexity of the interactions between the mammalian microbiome and its host, the use of model organisms such as the nematode worm *Caenorhabditis elegans* is a promising strategy to study host-microbiome interactions in vivo, as well as bacterial crosstalk within the host. Previously it was found that native bacterial isolates of the worm, *Chryseobacterium* sp. CHNTR56 MYb120 and *Comamonas* sp. 12022 MYb131, possess genomic diversity in the biosynthesis of the active form of vitamin B6, pyridoxal 5′-phosphate (PLP), and contribute to host fitness and lifespan extension. However, the relative contribution of PLP from each isolate, as well as the existence of interbacterial relationships within the worm gut remain to be characterized. In the present work, we investigated the presence and measured the abundance of PLP in the isolates and in the worms grown with the isolates using ultraperformance liquid chromatography tandem-mass spectrometry (UPLC-MS/MS). Our analyses confirmed the presence of PLP in vitro and in vivo. The elevated abundance of PLP in the isolates (which reached statistically significant levels when the two isolates were combined), and within worms grown with the combination of bacterial isolates, compared to control, indicated synergism between the isolates in the production of PLP. Isotope labeling revealed that *Comamonas* sp. 12022 MYb131 was the main provider of PLP in worms grown with the combination of bacterial isolates. The dominance of this isolate inside the worm was further confirmed by a colonization assay. An untargeted metabolomics analysis of the bacteria showed that the pathways related to cell growth, protein synthesis and lipid synthesis/energy production were regulated in the combination group in comparison with *Comamonas* sp. 12022 MYb131 alone. Furthermore, glutamine, involved in the *de novo* synthesis of purine and pyrimidines, was specifically abundant in this group, indicating the potential role of this metabolite in initiating and sustaining bacterial growth. This bacterial crosstalk is suggested to promote the growth of *Comamonas* sp. 12022 MYb131 in vivo, and synthesis of bacterial metabolites such as PLP in the worm gut.

## 1. Introduction

*Caenorhabditis elegans* is a nematode worm that has been utilized in many research studies since its introduction to science by Brenner [1]. This worm has been chosen by independent research groups because of various advantages with regard to its simple maintenance in standard laboratory conditions and its genetic similarities with higher eukaryotes including human. Although this nematode organism has been traditionally maintained with a standard laboratory bacterial strain, *Escherichia coli* OP50, wild *C. elegans* has its own associated microbiome containing four phyla-Bacteroidetes, Actinobacteria, Firmicutes and Proteobacteria as described in a recent study [2]. It has also been shown that this core set of the native microbiome colonize the worm’s gut, based on surface sterilization with a 4% bleach solution [3], and these phyla are also important members of the human gut microbiome [4]. 

To date, the effects of *Ochrobactrum* spp., belonging to Proteobacteria, on worm physiology have been analyzed globally with the use of omics technologies [5]. In this study, proteomic profiles (a total of 4677 proteins were identified) of worms grown with *Ochrobactrum* spp. revealed the modulation of host signaling pathways (such as DAF-2 insulin-like signaling pathway) and the immunological processing of foreign entities. Another study showed that *Ochrobactrum* isolates regulated *C. elegans* physiology through the metabolism of specific amino acids, fatty acids, and folate biosynthesis [6]. Despite these efforts, the biological influence of the vast majority of the native microbiome members of *C. elegans* remains to be demonstrated by the research community.

Evolutionarily, animals, including *C. elegans*, have lost critical genes such as *pdx* isomers (*pdxA*, *pdxB*, *pdxJ*), *SNZ* or *SNO* gene orthologs to synthesize vital molecules such as vitamin B6 (pyridoxal 5′- phosphate; PLP) [7] and, therefore, these organisms depend on their diet or microbiome for survival. The bacterial biosynthesis of the active form of vitamin B6 is known to be completed by *de novo* and/or salvage pathways through biochemical reactions based on activities of various enzymes (PDXJ/H/K/Y) [8,9]. In the *de novo* synthesis pathway, pyridoxine 5′-phosphate (PNP) is produced via a combination of 3-amino-1-hydroxyacetone 1-P (derived from D-erythrose 4-P) and D-xylulose 5-P (derived from D-glyceraldehyde 3-P + pyruvate) by PDXJ and converted to PLP by PDXH. In the salvage pathway, externally obtained pyridoxine, pyridoxamine or pyridoxal molecules can be converted to PLP. In this pathway, PNP can either be derived from pyridoxine by PDXK and converted to PLP by PDXH or pyridoxamine can be converted to pyridoxamine 5′-phosphate (PMP), which forms PLP (through subsequent actions of PDXK and PDXH). Additionally, pyridoxal can also be converted to PLP by PDXK/Y. Accumulated PLP can then be dephosphorylated and exported outside the bacterial cell by phosphatases such as PDXP [8,10]. In the de novo synthesis pathway, the production of 3-amino-1-hydroxyacetone 1-P triggers a specific route of the de novo pathway, but not D-erythrose 4-P production, which can be used for various other biological reactions [9,11]. 

Recently, the native bacterial isolates of the *C. elegans* microbiome were predicted to produce various metabolites (e.g., amino acids, vitamins) based on the bacterial genomic features [12]. These metabolites could play active roles in host-microbiome interactions. We have recently demonstrated that certain members of the *C. elegans* microbiome, *Chryseobacterium* sp. Both CHNTR56 MYb120 and *Comamonas* sp. 12022 MYb131, have genomic capacities for the synthesis of PLP and are beneficial to host fitness and longevity [13]. However, the presence and abundance of this critical vitamin in the bacterial isolates and worm have yet to be validated and characterized. The objective of this study was to determine the presence and abundance of PLP in the bacteria and their host using ultra-performance liquid chromatography tandem-mass spectrometry (UPLC-MS/MS), to profile metabolomes of the bacteria globally, as well as to investigate detailed mechanisms of interactions by using a labeled (^13^C) source for one isolate (*Chryseobacterium* sp. CHNTR56 MYb120) and an unlabeled source (^12^C) for the other (*Comamonas* sp. 12022 MYb131).

## 2. Results

### 2.1. Presence and Abundance of PLP in the Native Bacterial Isolates

**Figure 1:**
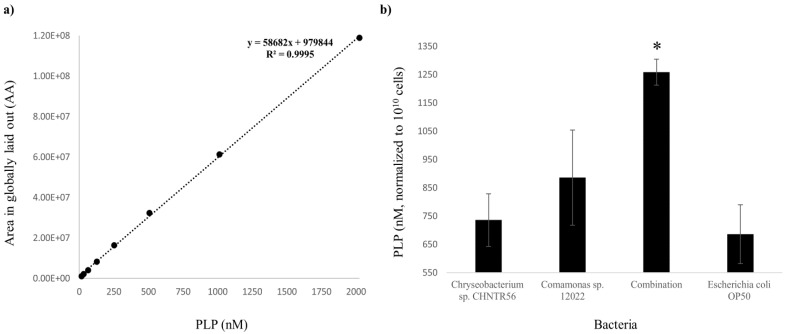
Abundance of PLP (nM) in bacterial isolates. (**a**) Linear curve of standard PLP with R^2^ score is shown; (**b**) The abundance of PLP (normalized to 10^10^ cells) was found to be the highest in the combined bacterial isolates. *: *p* < 0.05.

Although the presence of PLP in the isolates was predicted by Nanopore sequencing [13], it was not confirmed by analytical methods and so its abundance in the isolates remained unknown. In this study, the presence of PLP in bacteria was confirmed with UPLC-MS/MS analysis (Supplementary Figure S1). The detection of PLP ions in the samples was based on *m/z* value of standard PLP by MS analysis (a single peak at 248.0318) followed by MS/MS analysis (three major peaks at 94.0656, 122.0602 and 150.0550), at retention times ranging between 1.09 and 1.23 (among different runs throughout this study). Deviations in retention time (min) and mass errors (ppm) in *m/z* values of the MS/MS peaks, for each injection in an individual run, were around 0.02 and 0.0001, respectively. Minimum and maximum detectable levels of PLP were 0.49 and 8100 nanomolar (nM), respectively, and the curve derived from the dilutions of the standards demonstrated the robustness of the system (Supplementary Figure S2). The concentration of PLP was extrapolated from the external standard curve (R^2^ = 0.9995) (Figure 1a). The concentration of PLP (mean ± standard error) for each group were as follows: The abundance of PLP (mean ± standard error (SE)) showed a higher trend in *Chryseobacterium* sp. CHNTR56 MYb120 (735.73 ± 92.54 nM) or *Comamonas* sp. 12022 MYb131 (885.70 ± 168.08 nM), which reached a statistically significant level in the combined bacterial isolates (1258.42 ± 45.75 nM), compared to *E. coli* OP50 (685.56 ± 104.05 nM) (*p* < 0.05) (Figure 1b). 

Altogether, the data show that the combination of isolates synergistically promotes the biosynthesis of vitamin B6 (PLP) when they are grown together, initially from equal amounts of each isolate.

### 2.2. Presence and Abundance of PLP in the Worms Grown with the Native Bacterial Isolates

**Figure 2:**
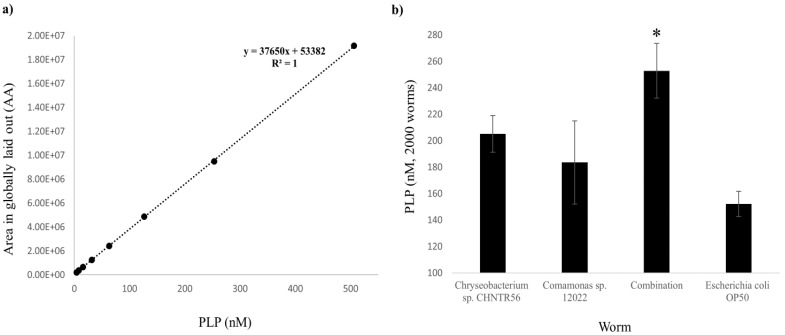
Abundance of PLP (nM) in worms grown with bacterial isolates. (**a**) Linear curve of standard PLP with R^2^ score is shown; (**b**) The abundance of PLP (from 2000 worms) was significantly higher in worms grown with the combination of bacterial isolates, while it was similar among worms grown with the isolates individually or *E. coli* OP50. *: *p* < 0.05.

The presence of PLP in worms grown with *Chryseobacterium* sp. CHNTR56 MYb120, *Comamonas* sp. 12022 MYb131, combination of both or *E. coli* OP50 was detected with UPLC-MS/MS analysis, based on *m/z* values at the MS and MS/MS levels as described in the previous section (Supplementary Figure S3). Minimum and maximum detectable levels of PLP did not change and the system robustness was maintained (Supplementary Figure S4). The concentration of PLP in worms was extrapolated from the external standard curve (R^2^ = 1) (Figure 2a) and normalized to the protein concentration of worms (determined by BCA assay). The abundance of PLP in worms grown with *Chryseobacterium* sp. CHNTR56 MYb120 (205.13 ± 13.80 nM) or *Comamonas* sp. 12022 MYb131 (183.51 ± 31.422 nM) was similar, while those grown with combination of both isolates was significantly higher (252.80 ± 20.75 nM) (*p* < 0.05), in comparison with those grown with *E. coli* OP50 (152.12 ± 9.55 nM) (Figure 2b).

In summary, these data are consistent with what is observed in the bacterial cultures, as the combination of isolates significantly promoted the biosynthesis of vitamin B6 (PLP) within the worm.

### 2.3. Contribution of PLP in the Worm from Each Individual Bacterium 

To understand the contribution of PLP from the native bacterial isolates in the worm, we first tested the presence of ^13^C-labeled PLP in all the bacteria studied. The presence of the labeled PLP, based on the predicted *m/z* values at the MS level (256.0587) and MS/MS level (100.0856, 129.0836 and 158.0818), was detectable in *Chryseobacterium* sp. CHNTR56 MYb120, combination and *E. coli* OP50, but not *Comamonas* sp. 12022 MYb131 (which does not prefer glucose as a sole carbon source for its growth [14] (Supplementary Figure S5)). The isotopologic masses of fully labeled PLP were predicted based on the following factors: (1) the *m/z* shift (MS and MS/MS levels) is approximately 8.0264 as PLP contains a total of eight carbon atoms [mass shift, 8 × 1.0033 Da (weight of a neutron)]; (2) the related peak at the indicated *m/z* value was observed in *Chryseobacterium* sp. CHNTR56 MYb120, combination and *E. coli* OP50, but not in *Comamonas* sp. 12022 MYb131; and (3) *m/z* values of major daughter ion in three carbon labeled PLP reported in a related study [15]. The isotope labeling strategy was successful in fully labeling PLP in *Chryseobacterium* sp. CHNTR56 MYb120, combination and *E. coli* OP50. 

Isotope labeling in the worms grown with the combination of these isolates demonstrated the contribution of PLP by *Comamonas* sp. 12022 MYb131 (fed on ^12^C glucose as a principal carbon source) and by *Chryseobacterium* sp. CHNTR56 MYb120 (fed on U-^13^C glucose as a principal carbon source). The peak of unlabeled PLP (derived from *Comamonas* sp. 12022 MYb131) with its daughter ions was detectable at the MS and MS/MS levels, respectively (Supplementary Figure S6). This was not the case for the peak of labeled PLP (derived from *Chryseobacterium* sp. CHNTR56 MYb120), which was not observed at the MS level in the worms grown with the combination of both isolates, indicating that PLP was mainly provided to the worm by *Comamonas* sp. 12022 MYb131. Based on the experimental factors in this study, the PLP of bacterial origin was assumed to be transmitted to the worm as (1) the only nutrient sources were bacteria, (2) the worm cannot synthesize PLP and (3) worms did not show insufficiency in terms of growth and motility. 

Minimum and maximum detectable levels of PLP (unlabeled) were the same as described previously. The concentration of PLP (unlabeled) was extrapolated from the external standard curve (R^2^ = 0.9999) (Supplementary Figure S7). The abundances of PLP (mean ± SE) derived from *Comamonas* sp. 12022 MYb131 and *E. coli* OP50 were estimated 43.39 ± 8.90 nM and 33.45 ± 2.10 nM, in the worm homogenates, respectively. The exact abundance of PLP from *Chryseobacterium* sp. CHNTR56 MYb120, in the worm homogenate, was not detectable (due to lack of peak area) at the minimum detection limit for PLP (0.49 nM). Additionally, the abundance of PLP from worms grown with both isolates or with *E. coli* OP50 was found to be approximately five times lower, compared to those grown under the conditions described previously in this text. Altogether, these data point to the possibility that either *Chryseobacterium* sp. CHNTR56 MYb120 does not produce PLP in the worm (in vivo) or its population is relatively low within the gut of *C. elegans*.

**Figure 3:**
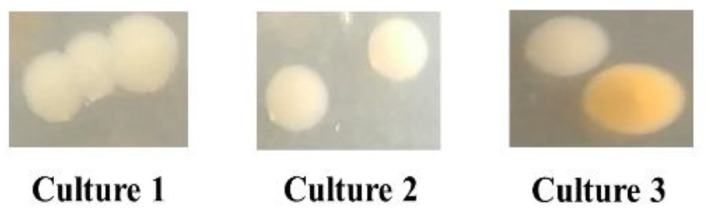
A depiction of recovered bacterial colonies from worms grown with both isolates. Majority of colonies (white colonies) belonged to *Comamonas* sp. 12022 MYb131, at a dilution factor of the worm homogenate at 10^−4^ dilution, which further supports the isotope-labeling findings. Orange colony represents *Chryseobacterium* sp. CHNTR56 MYb120. The images of the colonies were zoomed in on to show the bacterial color properly at the indicated dilution where bacterial colonies can be deciphered.

The colonization assay further confirmed the isotope labeling results, as the recovered colonies from worms grown with both isolates mainly belonged to *Comamonas* sp. 12022 MYb131 (Figure 3). The mean ± SE of the recovered bacterial colonies were as follows: 333.33 ± 210.77 CFU/worm for *Chryseobacterium* sp. CHNTR56 MYb120 and 2333.33 ± 333.26 CFU/worm for *Comamonas* sp. 12022 MYb131 for the worm group fed both isolates and 156.67 ± 43.86 CFU/worm for the worm group fed *E. coli* OP50. The SE was high for *Chryseobacterium* sp. CHNTR56 MYb120 as the colony formation of this bacterium was not observed in some replicates of cultures because of its low presence (Figure 3). This is in agreement with a recent report which states that *Chryseobacterium* sp. CHNTR56 MYb120 is a low colonizer of the nematode [3].

### 2.4. Metabolome Profiling of Bacteria at the Global Level

**Figure 4:**
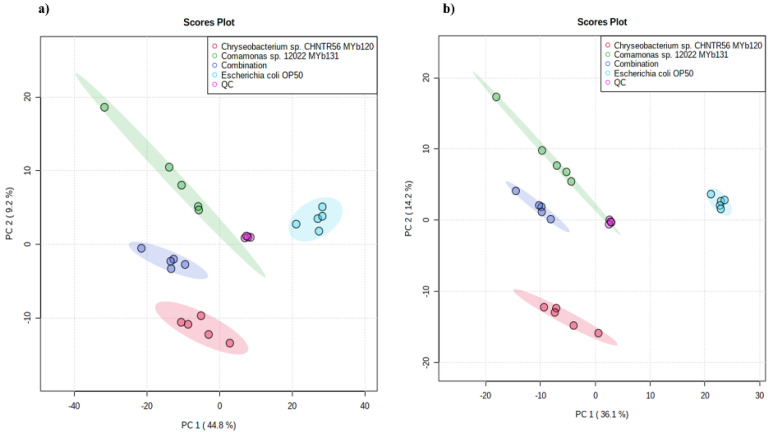
Principal component analysis (PCA). PCA demonstrates the similarity of the samples (*n* = 5 per group) based on their metabolite patterns for positive (**a**) and negative (**b**) modes. The QC samples are almost identical, indicating the robustness for each injection.

As PLP is promoted by the combination of bacterial isolates (*Chryseobacterium* sp. CHNTR56 MYb120 and *Comamonas* sp. 12022 MYb131), indicating bacterial crosstalk, we profiled the metabolomes of each bacterium and their combination to better understand the reason underlying this effect at the global level. When comparing all experimental groups (*Chryseobacterium* sp. CHNTR56 MYb120, *Comamonas* sp. 12022 MYb131, combination, *E. coli* OP50 or QC), a total of 3604 (positive ion mode) and 2161 (negative ion mode) features were obtained. A principal component analysis (PCA) demonstrated similar patterns of group separation in both the positive (Figure 4a) and negative modes (Figure 4b). 

**Figure 5:**
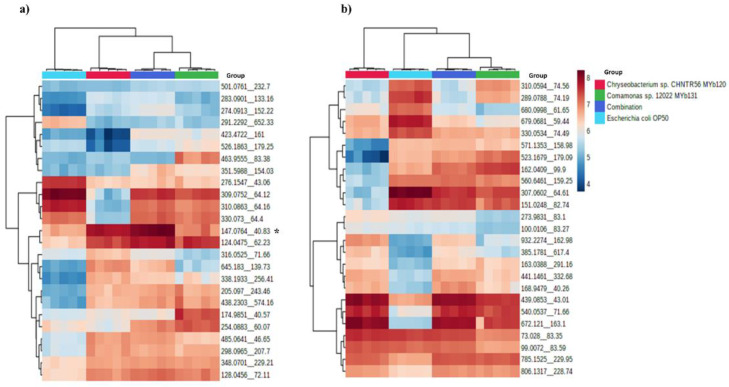
Heatmap of the top 25 significant features identified in positive (**a**) and negative (**b**) modes. The relative abundance of the feature (*m/z* = 147.0764), indicated with asterisk (*), is specifically promoted in the combination group in positive mode.

A total of 3156 and 1689 features were differentially presented in positive and negative modes (pair-wise comparison), respectively (Supplementary Table S2). A heatmap analysis showed distinct patterns among experimental groups. Figure 5 shows the top 25 significant features in both modes. A feature with *m/z* value of 147.076 in the positive mode was present in *Chryseobacterium* sp. CHNTR56 MYb120 at a higher relative abundance which was further increased in the combination group, but reduced in *Comamonas* sp. 12022 MYb131 (Figure 5a). 

We further performed a pathway analysis based on the *mummichog* method. The enriched pathways include purine metabolism, cysteine and methionine metabolism, pyrimidine metabolism, pantothenate and CoA biosynthesis, as well as pathways related to amino acid metabolism/protein synthesis (Table 1).

Comparing *Comamonas* sp. 12022 MYb131 with *Chryseobacterium* sp. CHNTR56 MYb120 or *E. coli* OP50, showed similarities in terms of enriched pathways. This is likely related to lower growth capacity of *Comamonas* sp. 12022 MYb131 in comparison with its counterparts. The enriched pathways for all group comparisons are shown in detail in Supplementary Table S3.

### 2.5. Identification and Quantification of Glutamine 

The feature of interest (*m/z* = 147.076) identified from the previous section was found to be present in the purine and pyrimidine metabolism pathways. It was predicted to be glutamine, based on the information from the *E. coli* metabolome database (Available online: https://ecmdb.ca/ (accessed on 4 October 2021)) [16]. As glutamine was observed to be most differentially present (based on peak intensity) in the comparison of *Comamonas* sp. 12022 MYb131 with combination (Figure 5a), as well as being an essential metabolite for de novo synthesis of purines and pyrimidines [17], it could play an important role in the inter-bacterial interactions. 

**Figure 6:**
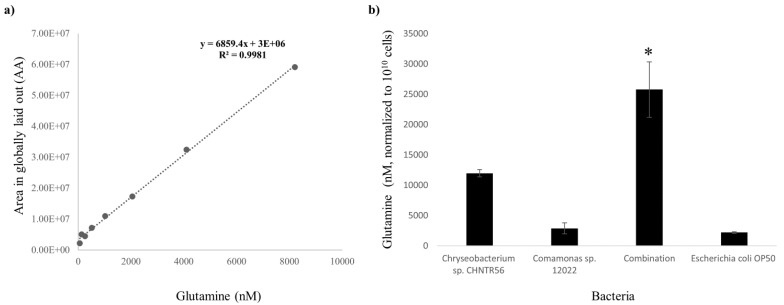
Abundance of glutamine in bacterial isolates. (**a**) Linear curve of standard glutamine (*m/z* = 147.0764) with R^2^ score is shown; (**b**) The abundance of glutamine (normalized to 10^10^ cells) was estimated to be the highest in combination. *: *p* < 0.05.

The presence of glutamine was confirmed by an LC-MS/MS analysis (Supplementary Figure S8). The detection of glutamine in the bacterial samples was based on *m/z* value and retention time of standard glutamine by MS (a single peak at 147.0764) followed by MS/MS (three peaks at 84.0449, 101.0712 and 130.0499). Deviations in retention time and in *m/z* values of the MS/MS peaks, for each injection in an individual run, were 0.01 and 0.0001, respectively. Minimum and maximum detectable levels of glutamine were estimated to be 64.14 and 8210 nanomolar (nM), respectively. Concentration of glutamine was extrapolated from the external standard curve (R^2^ = 0.9981) (Figure 6a). The abundance of glutamine (mean ± SE) was estimated highest in combination (25,776.76 ± 4569.81 nM), in comparison with *Chryseobacterium* sp. CHNTR56 MYb120 (11,949.25 ± 605.06 nM), *Comamonas* sp. 12022 MYb131 (2864.65 ± 908.68 nM) or *E. coli* OP50 (2187.62 ± 143.63 nM) (*p* < 0.05) (Figure 6b), consistent with the finding for this metabolite in untargeted global metabolomic analysis (Figure 6b). 

These results indicate that glutamine is an important metabolite involved in the crosstalk between *Chryseobacterium* sp. CHNTR56 MYb120 and *Comamonas* sp. 12022 MYb131.

## 3. Discussion

In our previous study, we demonstrated that *Comamonas* sp. 12022 MYb131 and *Chryseobacterium* sp. CHNTR56 MYb120 have multiple gene homologs in the biosynthesis of PLP and positively influence host fitness and longevity [13]. In addition to this, transcriptomic changes in worms grown with the indicated isolates demonstrated an upregulation of host cysteine synthases (*cysl* genes) which implied the importance of PLP (co-factor of *cysl* genes). Since the worm does not synthesize PLP, its supply should be from bacterial diet or the gut microbiome. 

In the present study, we investigated whether the native bacterial isolates of *C. elegans* synthesize PLP and, if so, what the abundance of PLP in the worm and the contribution of PLP by each isolate is. As we used minimal media, PLP was assumed to be produced by bacteria (in vitro), and PLP extracted from worms was only of bacterial origin (in vivo) because there was no additional source of PLP in the related experiments in our study, while *C. elegans* lacks the necessary enzymes for its production. Additionally, worm growth and motility were not affected, which also suggested that vital bacterial nutrients that the worm cannot synthesize (e.g., vitamins such as PLP) was successfully transmitted to the worm from bacteria (grown in minimal media).

We found that *Comamonas* sp. 12022 MYb131 and *Chryseobacterium* sp. CHNTR56 MYb120 synthesize PLP, and its abundance is promoted when isolates are grown together, initially from an equal amount of each isolate, and in the worms grown with the combination of the isolates, compared to control (*p* < 0.05). This effect could be the result of an event where isolates influence each other to increase PLP production synergistically or one bacterial isolate becomes dominant and produces most of this essential metabolite, through the influence of the other bacterium. To test this, we monitored the contribution of PLP from each isolate using isotope labeling and the abundance of live bacteria in the worms grown with the combined isolates using a colonization assay. 

The isotope labeling strategy, where PLP was differentially labeled depending on the isolate, demonstrated that PLP derived from *Comamonas* sp. 12022 MYb131 (^12^C) was prominent (based on the peak which was detected at 248.0318 at the MS level), but not for labeled PLP derived from *Chryseobacterium* sp. CHNTR56 MYb120 (^13^C), indicating PLP is predominantly obtained from *Comamonas* sp. 12022 MYb131 in the worms grown with the combination of these isolates. As there was a possibility that *Chryseobacterium* sp. CHNTR56 MYb120 can provide PLP within the worm, we performed a colonization assay to determine the relative population of both isolates within the worm.

A recovery of live bacteria in the worms by the colonization assay supported the isotope-labeling results. This analysis revealed that the recovered live bacteria in worms grown with the combined isolates were mainly *Comamonas* sp. 12022 MYb131. Both the isotope labeling and the colonization assay suggested that *Comamonas* sp. 12022 MYb131 is the principal source of PLP, which appeared to be regulated by the presence of *Chryseobacterium* sp. CHNTR56 MYb120 in the worm. 

To further investigate metabolome profiles of bacterial isolates, we carried out untargeted metabolomics. This analysis showed distinct patterns for each bacterium or their combination. A feature at a *m/z* value of 147.0764, confirmed to be glutamine, was promoted in the combination of isolates (where *Comamonas* sp. 12022 MYb131 overpopulate) but decreased in *Comamonas* sp. 12022 MYb131 alone. A pathway analysis showed that glutamine participating pathways such as purine and pyrimidine metabolisms (involved in bacterial cell proliferation [18]) were found to be differentially regulated. The other regulated biological pathways, such as cysteine and methionine metabolism (involved in protein synthesis) [19] and pantothenate and CoA biosynthesis (involved in energy the synthesis of phospholipids, synthesis/degradation of fatty acids, and energy production through the operation of the tricarboxylic acid cycle) [20,21] appear to complement this process. Overall, these findings indicate that *Chryseobacterium* sp. CHNTR56 MYb120 might directly provide glutamine to *Comamonas* sp. 12022 MYb131 by establishing a suitable environment (e.g., influencing environmental pH) for glutamine and/or glutamic acid transports across the bacterial cell wall, and/or upregulations of the related genes of *Comamonas* sp. 12022 MYb131 for glutamine synthesis through signaling molecule(s) of *Chryseobacterium* sp. CHNTR56 MYb120 origin, which initiates and maintains bacterial proliferation. 

The current study has its limitations. Firstly, *Comamonas* sp. 12022 MYb131 and *Chryseobacterium* sp. CHNTR56 MYb120 have natural diversity in utilizing nutrients (carbohydrates in particular) and differences in growth. Secondly, during isotope labeling, bacteria were seeded on peptone-free plates (to minimize ^12^C related sources), leading to low PLP levels in worms grown on these bacteria, which could be related to reduced growth and hence, the metabolism of bacteria. We will take into account these challenges in the design of our future research.

## 4. Materials and Methods

### 4.1. Caenorhabditis Elegans and Bacterial Species

The *C. elegans* N2 strain (Bristol) and *Escherichia coli* OP50 were obtained from the Caenorhabditis Genetics Center (CGC) at the University of Minnesota. Two native bacterial isolates, *Chryseobacterium* sp. CHNTR56 MYb120 and *Comamonas* sp. 12022 MYb131, were kindly provided by Prof. Hinrich Schulenburg, Zoological Institute, Evolutionary Ecology and Genetics, Christian-Albrecht University of Kiel, Kiel, Germany.

### 4.2. Worm Maintenance and Bacterial Culture

*Escherichia coli* OP50 and each native bacterial isolate, from their frozen stocks, were grown in at 37 °C and at 28 °C in tryptic soy agar (TSA) plates, respectively. The N2 strain was maintained at 21 °C in an incubator on nematode growth media (NGM) plates using *E. coli* OP50. The nematode worms were synchronized using 16.6% alkaline bleach (*v/v*) to kill the adults and to obtain eggs, so as to prevent any bacterial contamination. After two initial washings with M9 buffer, eggs were separated from the dead worm carcasses by centrifugation on a 60% sucrose (*w/v*) gradient at 400× *g* for 6 min at room temperature. The eggs were then washed two times with M9 buffer and were left rocking overnight in M9 buffer to hatch into the L1 stage.

### 4.3. Detection and Measurement of PLP in Bacterial Isolates

In this and subsequent sections, samples from each experiment were prepared at the same time point under the same laboratory conditions and subjected to UPLC-MS/MS within a single run. *Escherichia coli* OP50 and the native bacterial isolates, either individually or combined, were grown at 37 °C and at 28 °C in minimal growth media containing 0.2% glucose, 1 µM MgSO_4_, 0.05 µM CaCl_2_ and 0.1% casamino acids, in triplicate, respectively. Sample preparation for UPLC-MS/MS was based on a previously described method [22]. Bacterial isolates were centrifuged (4000 rpm for 10 min at 4 °C), the supernatant was decanted, and the bacterial pellet was quenched with liquid nitrogen and stored at −80 °C until extraction. The frozen pellet was mixed with a total of 500 µL methanol and subjected to freeze–thawing followed by vortexing for 60 s. Methanol was evaporated using a vacuum concentrator and the dried content was resuspended with 160 µL of water containing 0.1% formic acid (*v/v*). The resuspension was filtered using a centrifugal filter (0.2 µm) at 14,000× *g* for 15 min at 4 °C and transferred to MS vials. The filtered samples were then run in UPLC-MS/MS. Dilutions of external standard (PLP from 8100 nM to 0.49 nM, 2-fold) were prepared in water containing 0.1% formic acid (*v/v*). The UPLC-MS/MS measurement was performed using an Ultimate 3000 liquid chromatography coupled with Q-Exactive Orbitrap HRMS (Thermo Fisher Scientific, Waltham, MA, USA). A chromatographic separation was performed on Thermo Hypersil GOLD C18 column (100 mm × 2.1 mm, 1.9 µm), with a flow rate of 0.4 mL/min. The injection volume was 5 μL. The gradient elution was composed of mobile A phase (0.1% formic acid in water) and mobile phase B (0.1% formic acid in acetonitrile) and performed for a 12 min run as shown in Supplementary Table S1. The Q-Exactive Orbitrap HRMS was equipped with heated electrospray ionization (HESI) source using the following source parameters: sheath gas flow: 55 arb (arbitrary units); aux gas flow: 10 arb; spray voltage: + 4.0 KeV, capillary temperature: 350 °C; S-lens RF level: 55; aux gas heater temperature: 300 °C. Positive ion analysis was performed in the parallel reaction monitoring (PRM) mode. The target mass-to-charge ratio (*m/z*) for PLP was 248.0318 as determined by using commercially available standard for PLP. Peak area of PLP for each sample and for the external standard were calculated using the global function in the FreeStyle software (v1.6, Thermo Scientific). The abundance of PLP (nanomolar; nM) for each sample was extrapolated from the curve generated by external standard and further calculated by normalization factors which are based on bacterial abundance derived from OD_595nm_ values using an online resource (Available online: http://www.labtools.us (accessed on 4 October 2021)) optimized for *E. coli*, which shares similarity in shape (rod shape) with other bacteria used in this study.

### 4.4. Detection and Measurement of PLP in Worms Grown with the Native Bacterial Isolates or E. coli OP50

Bacteria were grown as described in the previous section. Equal concentrations of *E. coli* OP50, each native bacterial isolate or combination of both, plated at a final OD_595_ = ~ 5, were seeded on NGM plates and incubated at 37 °C (for *E. coli* OP50) or at 28 °C (for native bacterial isolates) about 24 h. Synchronized L1 worms (*n* = 2000) were grown to young adult worm stage, in triplicate, on NGM plates at 21 °C. Worms were then washed off from the plates using M9 buffer and the collected worms were centrifuged at 400× *g* for 3 min at room temperature to remove the supernatant. Worms were quenched with liquid nitrogen and stored at −80 °C until analysis. The content was mixed with 80% methanol (*v/v*), vortexed and transferred to ceramic-bead-containing tubes and homogenized using a bead beater (placing the sample on ice after every 30 s of homogenization, 5 times in total). The homogenate was centrifuged at 14,000× *g*, 4 °C, for 10 min and the supernatant was transferred to new tubes (1.5 mL) and evaporated using a vacuum concentrator. The prepared samples and external standard were subjected to UPLC-MS/MS analysis using the same LC-MS parameters and values. The abundance of PLP for each sample was measured as described in the previous section, except the worm protein concentration, measured with a commercial kit (Pierce Rapid Gold BCA Protein Assay Kit, Thermo Scientific), was used to normalize the data.

### 4.5. Isotope Labeling

Firstly, *E. coli* OP50 and each native bacterial isolate were grown in minimal growth media containing 0.2% labeled glucose (^13^C), 1 µM MgSO_4_, 0.05 µM CaCl_2_ and 0.1% casamino acids, at 37 °C and at 28 °C, respectively, to test the presence of labeled PLP. *Escherichia coli* OP50 and *Comamonas* sp. 12022 MYb131 were individually grown in minimal growth media containing unlabeled glucose (^12^C) and *Chryseobacterium* sp. CHNTR56 MYb120 was grown in minimal growth media containing labeled glucose (^13^C) to differentiate between the supply of PLP (unlabeled form from *Comamonas* sp. 12022 MYb131 and labeled form from *Chryseobacterium* sp. CHNTR56 MYb120). To minimize non-specific carbon sources, casamino acids were not included in the minimal growth media. Synchronized L1 worms (*n* = 1500) were grown with the combination of *Comamonas* sp. 12022 MYb131 and *Chryseobacterium* sp. CHNTR56 MYb120 (50%:50% relative abundance) or on *E. coli* OP50 alone to the young adult stage on equal concentrations of bacteria based on OD_595nm_ (final OD_595nm_ = 10), on peptone free NGM plates, in triplicate. Extraction of metabolites from worms was carried out as previously described and samples were analyzed with the set UPLC-MS parameters as described, except the *m/z* of 256.0587 was targeted to detect ^13^C labeled PLP. The abundance of PLP in worms was measured based on the peak area as described in the previous section.

### 4.6. Colonization Assay

Bacterial colonization inside the N2 strain worm was evaluated based on a previously published method [23]. Worms were grown to young adult worms, as described in the isotope-labeling section, except only unlabeled glucose (^12^C) was used. For the surface sterilization of the worms, the young adult worms grown with the combination of bacterial isolates (*n* = 5) were transferred to kanamycin NGM agar plates (50 µg/mL) and kanamycin (50 µg/mL) and ampicillin NGM agar plates (100 µg/mL), for 1 h for each antibiotic treatment (two-hour incubation in total), and those grown with *E. coli* OP50 (*n* = 5) were transferred to NGM ampicillin plates (100 µg/mL) for the same time period, in triplicate. The worms were transferred to a sterile M9 buffer and disrupted, after which the diluted content (10^−4^ dilution factor) was placed on TSB plates (in triplicates) to allow for bacterial colony growth. The number of bacterial colonies were reported as colony-forming units per worm (CFU/worm) after two days of growth on Tryptic Soy Agar (TSA) plates.

### 4.7. Global Metabolomics of Bacteria

To understand the difference in metabolism between the individual bacterial isolates and combination at the global level we performed untargeted metabolomics UPLC-MS analysis. Bacteria (*Chryseobacterium* sp. CHNTR56 MYb120, *Comamonas* sp. 12022 MYb131, combination of both or *E. coli* OP50) were grown and samples (five replicates per group) were prepared and subjected to UPLC-MS as described in the previous section (detection and measurement of PLP in bacterial isolates) except quality control (QC) (prepared by combining equal volumes of each sample from all the groups in four replicates) was used instead of external PLP standard. Positive and negative ion modes were used. Raw data format was converted to mzML format using ProteoWizard [24] and analyzed using MetaboAnalyst 5.0 [25]. For statistical analysis, sample normalization was performed by considering bacterial number (based on OD_595nm_) and data transformation was carried out with log 10 transformation. A principal component analysis (PCA) was applied to examine overall sample distribution, and ANOVA with Fisher’s Least Significant Difference (LSD) test was used to evaluate group difference. For functional analysis, the ion mode was chosen accordingly (positive or negative) and mass tolerance was set 5 ppm. The Mummichog algorithm [26] was used with default *p*-value cutoff and the data was visualized as heatmap and enriched pathways were reported. Pathway library of *Escherichia coli* K-12 MG1655 (KEGG; Kyoto Encyclopedia of Genes and Genomes; Available online: https://www.genome.jp/kegg/ (accessed on 9 September 2021)) was used.

### 4.8. Detection and Measurement of Glutamine in Bacterial Isolates

Bacteria (*Chryseobacterium* sp. CHNTR56 MYb120, *Comamonas* sp. 12022 MYb131, combination of both or *E. coli* OP50) were grown and samples (five replicates per group) were prepared and analyzed with UPLC-MS/MS as described in the previous section (detection and measurement of PLP in bacterial isolates) and subjected to UPLC-MS/MS analysis. The positive mode was used. The target mass/charge ratio (*m/z*) for glutamine was 147.0764, as determined by using the commercially available standard for glutamine. Detection and measurement of glutamine (nM) for each sample was estimated as described in Section 4.3. 30% of actual nM of external standard was used to generate the curve due to glutamine loss in the ionization source, which was previously described for this metabolite [27].

### 4.9. Statistical Analysis

The group differences in PLP and glutamine levels were examined by ANOVA with Fisher’s LSD test in R (version 4.0.2) [28].

## 5. Conclusions

The aim of this study was to investigate the presence and abundance of PLP in the isolates and the worms grown using the isolates and employing the associated analyses to better understand host-microbiome interactions in *C. elegans*. We demonstrated that the abundance of PLP is promoted by the combination of the bacterial isolates either in vitro or in vivo, and PLP is mainly derived from *Comamonas* sp. 12022 MYb131, in worms grown with these isolates together. The dominance of *Comamonas* sp. 12022 MYb131 along with elevated PLP levels in these worms suggest that *Chryseobacterium* sp. CHNTR56 MYb120 is the inducing factor that promotes PLP production, which is predominantly provided by the colonizing *Proteobacteria* species, *Comamonas* sp. 12022 MYb131, within the worm. The untargeted metabolomics analysis of bacteria further demonstrated that this interaction is indeed associated with bacterial growth through biological pathways, including purine and pyrimidine metabolism, which is linked to the presence of glutamine in the combination of isolates. This works presents novel findings on host-microbiome interactions as well as inter-bacterial crosstalk.

## Figures and Tables

**Table 1:** Enriched pathways (*p* value < 0.05) for comparison between *Comamonas* sp. 12022 MYb131 and combination. The enriched pathways are mainly identified from positive mode. Pathway names ending with (N) indicate that the results are based on negative modes.

KEGG Pathways	Hits (Total)	Hits (Sig)	*p*-Value (Gamma-Adjusted)
Purine metabolism	24	8	0.02868
Aminoacyl-tRNA biosynthesis	17	7	0.01341
Cysteine and methionine metabolism	13	5	0.01839
Pyrimidine metabolism	12	6	0.00892
Pantothenate and CoA biosynthesis	11	6	0.00772
Glutathione metabolism	7	4	0.00872
Lysine biosynthesis	6	4	0.00726
Glycine, serine and threonine metabolism	6	3	0.01255
Thiamine metabolism	6	3	0.01255
Histidine metabolism	6	2	0.03089
Sulfur metabolism	6	2	0.03089
Cyanoamino acid metabolism	5	3	0.00949
Tyrosine metabolism	5	2	0.02224
Riboflavin metabolism	5	2	0.02224
Purine metabolism (N)	15	8	0.01759
Galactose metabolism (N)	6	3	0.0283
Arginine and proline metabolism (N)	6	3	0.0283

## Data Availability

Data supporting the results are provided as supporting information (Tables S1–S3 and Figures S1–S8). All data can be available from authors upon reasonable request. The data are not publicly available due to its usage in an ongoing study.

## Supplementary Materials

The following supporting information can be downloaded at: https://www.mdpi.com/article/10.3390/metabo12020172/s1, Table S1: Gradient elution for LC. The percent of mobile phase B at each indicated time point for LC is shown. *: Equilibration, Table S2: Differentially presented metabolites. Differentially presented metabolites among groups (FDR < 0.05), determined by ANOVA with Fisher’s LSD, are shown, for both modes, Table S3: Enriched pathways. KEGG pathways that are enriched in two group comparisons are provided in detail. (N): Negative mode, Figure S1: Presence of PLP in bacteria. PLP in bacteria was confirmed with LC-MS/MS analysis where the *m/z* value of parent ion is 248.0318 and the *m/z* values of major daughter ions (±0.0001) are: 94.0656, 122.0602 and 150.0550, at RT:1.11 ± 0.02. MS with MS/MS spectra are shown for the combination group as representative. RT: retention time in minutes, Figure S2: Standard curve including the lowest and highest detectable levels of PLP (for the run measuring PLP in bacteria), Figure S3: Presence of PLP in worms grown with bacteria. PLP in worms (*n* = 2000) grown with *Chryseobacterium* sp. CHNTR56 MYb120, *Comamonas* sp. 12022 MYb131, combination of both or *E. coli* OP50 was confirmed based on MS spectra (*m/z* value = 248.0318) and MS/MS spectra [*m/z* values (±0.0002) = 94.0655, 122.0601 and 150.0549), at RT:1.12 ± 0.02]. MS with MS/MS spectra are shown for the combination group as representative, Figure S4: Standard curve including the lowest and highest detectable levels of PLP (for the run measuring PLP in worms), Figure S5: MS spectra and MS/MS spectra of ^13^C labeled PLP in bacteria. The presence of ^13^C labeled PLP is apparent in *Chryseobacterium* sp. CHNTR56 MYb120, combination and in *E. coli* OP50 at the predicted *m/z* values for MS (256.0587) and for MS/MS [*m/z* values (±0.0001) = 100.0856, 129.0836 and 158.0818)], at RT:1.11 ± 0.02, but not for *Comamonas* sp. 12022 MYb131. MS with MS/MS spectra are shown for the combination group as representative, Figure S6: Contribution of PLP by each native bacterial isolate in worms grown with both isolates. ^12^C and ^13^C derived PLP in worms grown with the combination of *Comamonas* sp. 12022 MYb131 and *Chryseobacterium* sp. CHNTR56 MYb120 were targeted. The expected *m/z* values at the MS level (248.0318) and MS/MS level (±0.0001), which are 94.0656, 122.0603 and 150.0551, for ^12^C derived PLP are detectable (at RT:1.17 ± 0.01), while the predicted *m/z* value at the MS level (256.0587) for ^13^C derived PLP is not prominent as a peak at any retention time point, indicating PLP is mainly provided by *Comamonas* sp. 12022 MYb131, Figure S7: External standard curve. Standard curve including nM concentration of PLP at globally laid peak areas is shown. Figure S8: Presence of glutamine in bacteria. Glutamine in bacteria was confirmed with LC-MS/MS analysis where the *m/z* value of parent ion is 147.0764, with the *m/z* values of major daughter ions (±0.0001), which are: 84.0449, 101.0712 and 130.0499, at RT:0.67 ± 0.01. MS with MS/MS spectra are shown for the combination group as representative.
