# Supplementary material for: Native Microbiome Members of C. elegans Act Synergistically in Biosynthesis of Pyridoxal 5′-Phosphate
Source: Metabolites. 2022 Feb 11;12(2):172. doi: 10.3390/metabo12020172 (PMC8879373; doi:10.3390/metabo12020172)
Supplement: Supplementary file 1 [file metabolites-12-00172-s001.zip › Supplementary Figures.pdf]

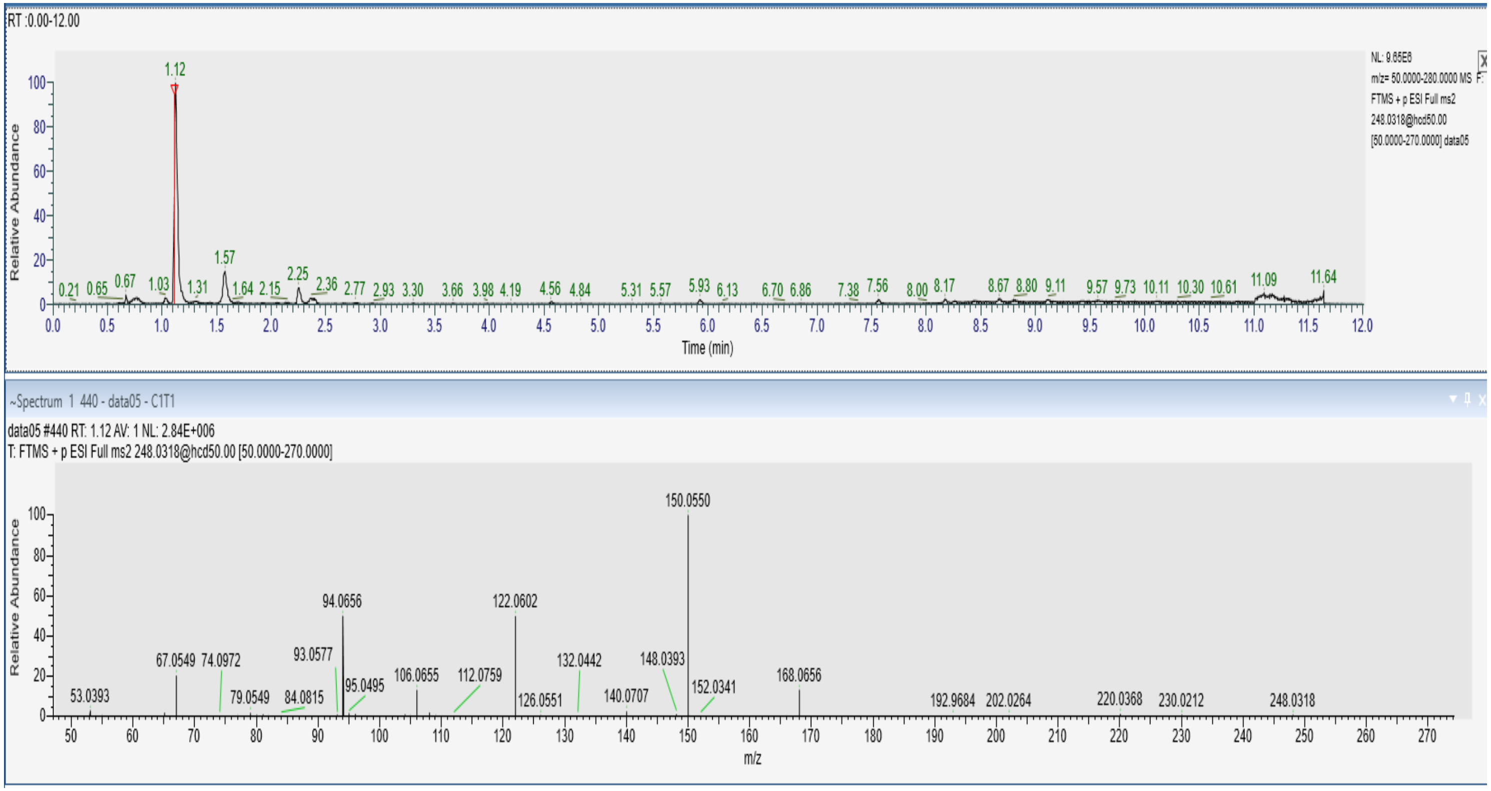

Figure S1: Presence of PLP in bacteria. PLP in bacteria was confirmed with LC-MS/MS analysis where the *m/z* value of parent ion is 248.0318 and the *m/z* values of major daughter ions (±0.0001) are: 94.0656, 122.0602 and 150.0550, at RT:1.11±0.02. MS with MS/MS spectra are shown for the combination group as representative. RT: retention time in minutes.

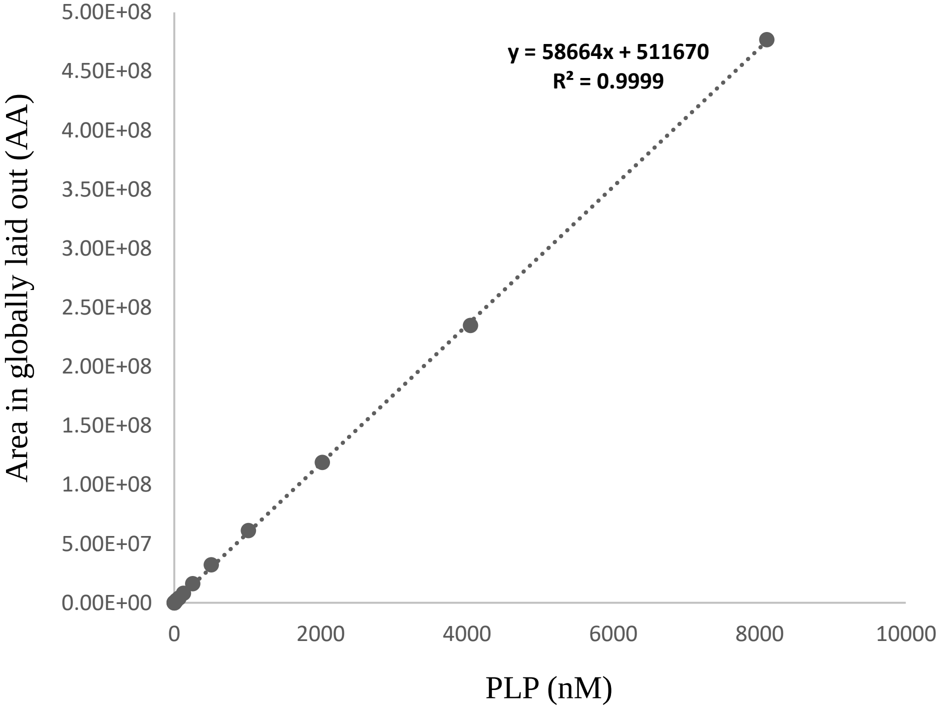

Figure S2: Standard curve including the lowest and highest detectable levels of PLP (for the run measuring PLP in bacteria).

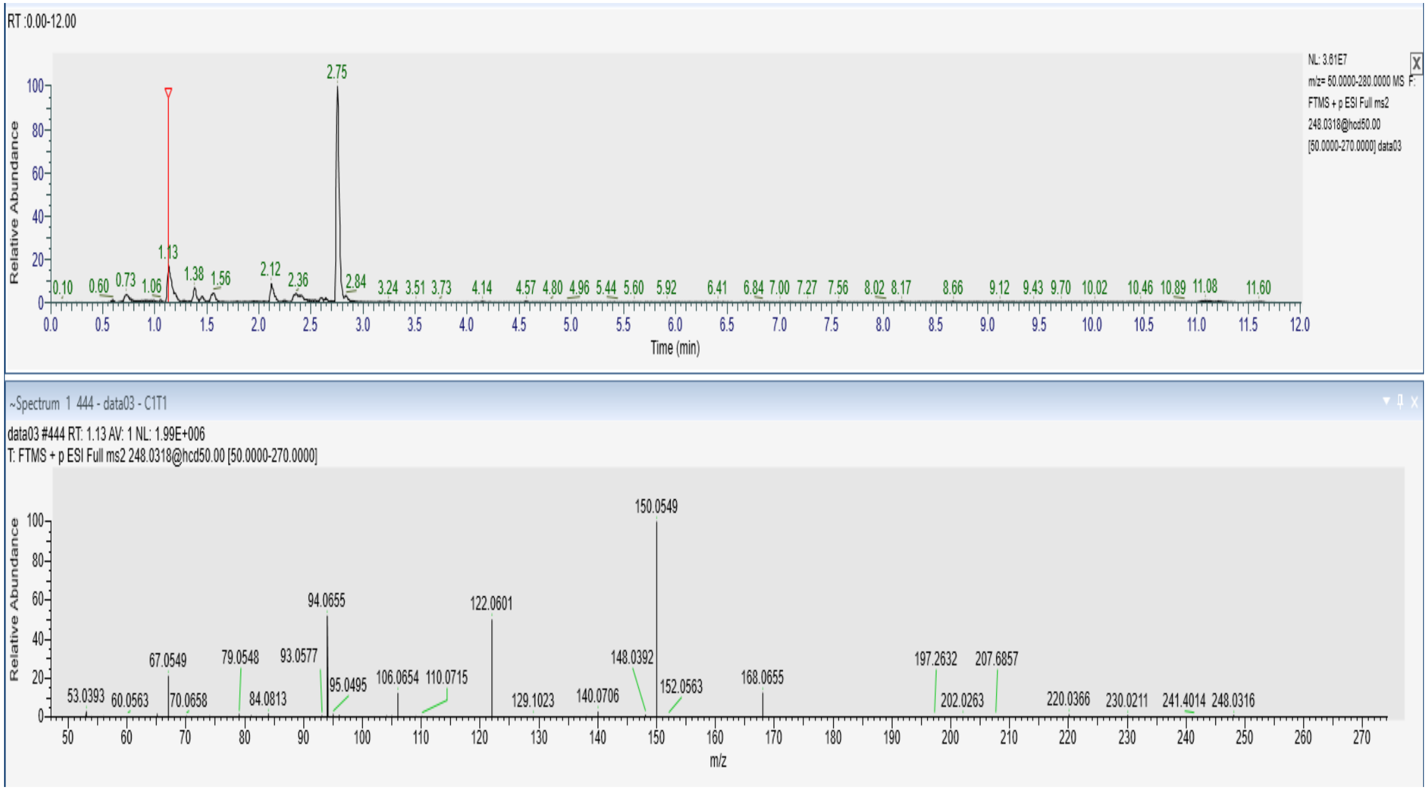

Figure S3: Presence of PLP in worms grown with bacteria. PLP in worms (*n*=2,000) grown with *Chryseobacterium* sp. CHNTR56 MYb120, *Comamonas* sp. 12022 MYb131, combination of both or *E. coli* OP50 was confirmed based on MS spectra (*m/z* value = 248.0318) and MS/MS spectra [m/z values (±0.0002) = 94.0655, 122.0601 and 150.0549), at RT:1.12±0.02]. MS with MS/MS spectra are shown for the combination group as representative.

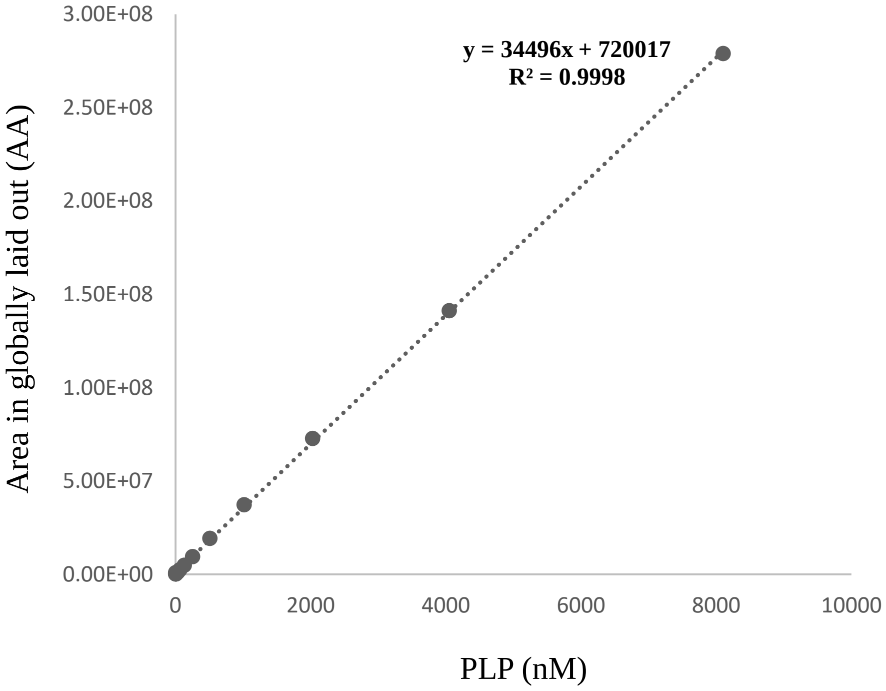

Figure S4: Standard curve including the lowest and highest detectable levels of PLP (for the run measuring PLP in worms).

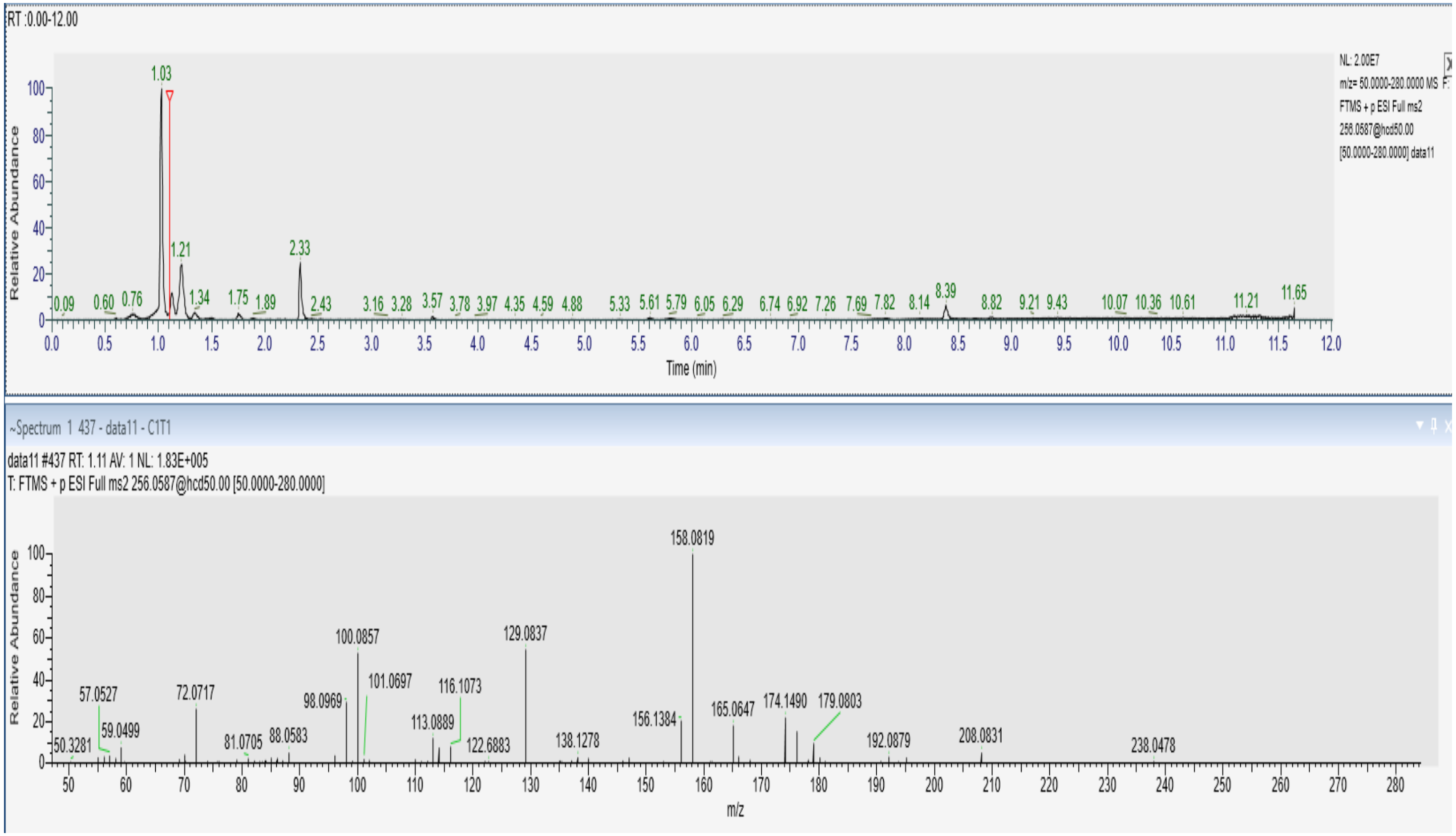

Figure S5: MS spectra and MS/MS spectra of ^{13}C labeled PLP in bacteria. The presence of ^{13}C labeled PLP is apparent in *Chryseobacterium* sp. CHNTR56 MYb120 and in *E. coli* OP50 at the predicted *m/z* values for MS (256.0587) and for MS/MS [m/z values ($\pm$0.0001) = 100.0856, 129.0836 and 158.0818)], at RT:1.11$\pm$0.02, but not for *Comamonas* sp. 12022 MYb131. MS with MS/MS spectra are shown for the combination group as representative.

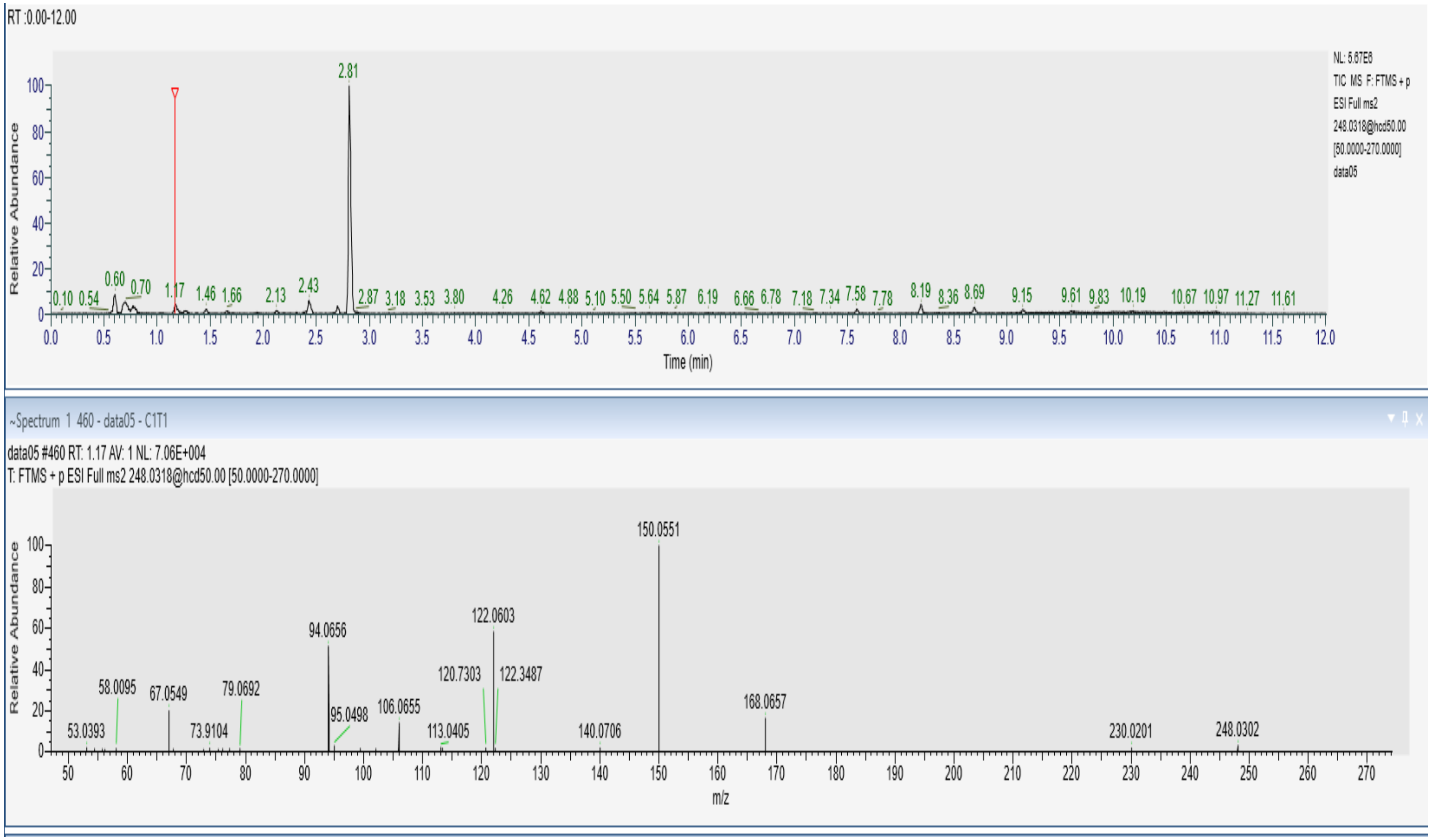

Figure S6: Contribution of PLP by each native bacterial isolate in worms grown with both isolates. ^{12}C and ^{13}C derived PLP in worms grown with the combination of *Comamonas* sp. 12022 MYb131 and *Chryseobacterium* sp. CHNTR56 MYb120 were targeted. The expected *m/z* values at the MS level (248.0318) and MS/MS level ($\pm$0.0001), which are 94.0656, 122.0603 and 150.0551, for ^{12}C derived PLP are detectable (at RT:1.17$\pm$0.01), while the predicted *m/z* value at the MS level (256.0587) for ^{13}C derived PLP is not prominent as a peak at any retention time point, indicating PLP is mainly provided by *Comamonas* sp. 12022 MYb131.

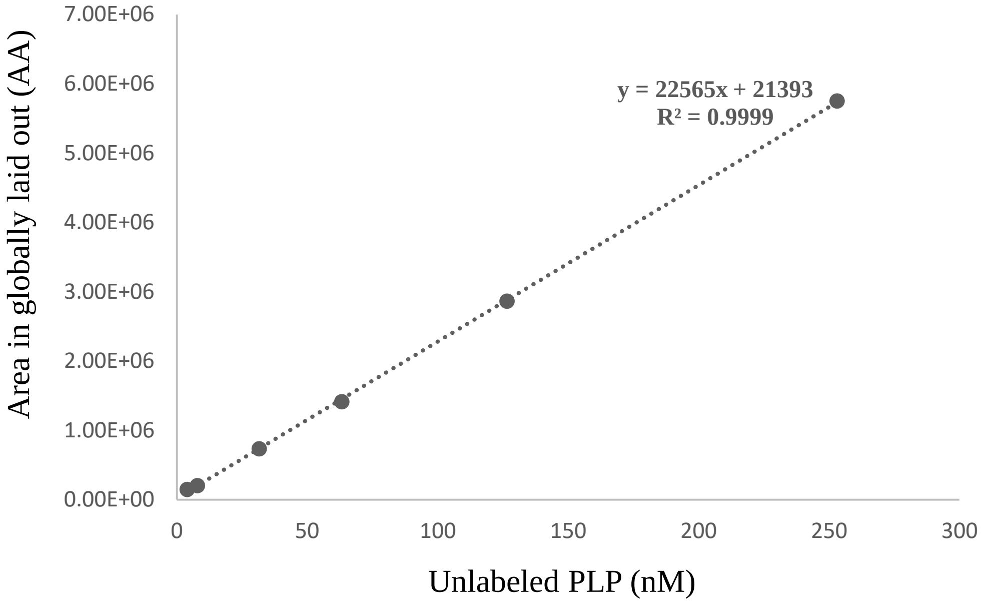

Figure S7: External standard curve. Standard curve including nM concentration of PLP at globally laid peak areas is shown.

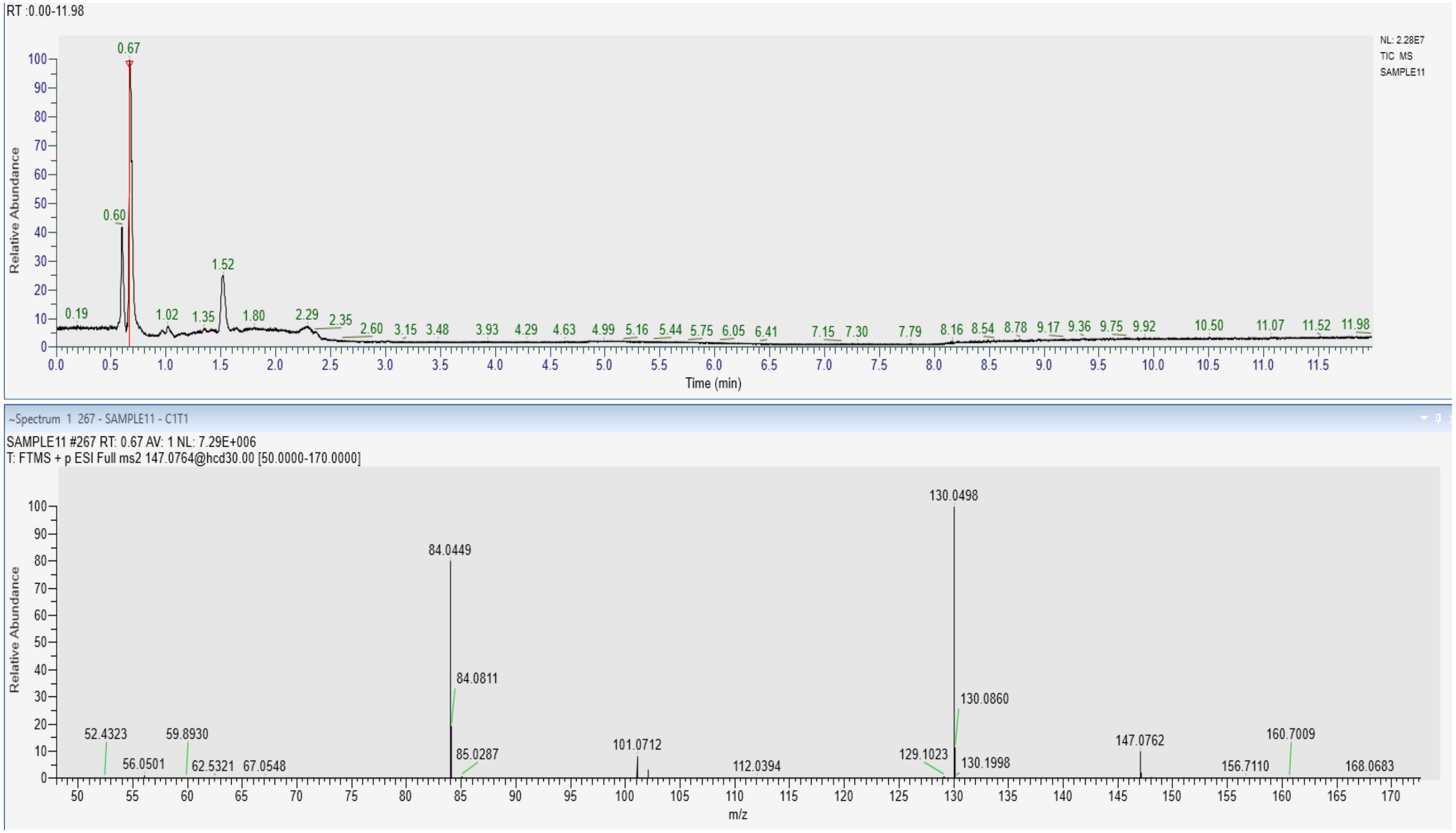

Figure S8: Presence of glutamine in bacteria. Glutamine in bacteria was confirmed with LC-MS/MS analysis where the *m/z* value of parent ion is 147.0764, with the *m/z* values of major daughter ions (±0.0001), which are: 84.0449, 101.0712 and 130.0499, at RT:0.67±0.01. MS with MS/MS spectra are shown for the combination group as representative.
